# Anti-Irritant Strategy against Retinol Based on the Genetic Analysis of Korean Population: A Genetically Guided Top–Down Approach

**DOI:** 10.3390/pharmaceutics13122006

**Published:** 2021-11-25

**Authors:** Seongsu Kang, Kyunghoe Kim, Seung-Hyun Jun, Seonju Lee, Juhyun Kim, Joong-Gon Shin, Yunkwan Kim, Mina Kim, Sun-Gyoo Park, Nae-Gyu Kang

**Affiliations:** Department of Core Technology, LG Household and Health Care R&D Center, Seoul 07795, Korea; franck.kang@lghnh.com (S.K.); kyunghkim93@lghnh.com (K.K.); seonju@lghnh.com (S.L.); juhyunkim@lghnh.com (J.K.); sssg001@lghnh.com (J.-G.S.); kimyoonkwan@lghnh.com (Y.K.); mnkim@lghnh.com (M.K.); skparke@lghnh.com (S.-G.P.)

**Keywords:** retinol, retinoid, cosmetics, anti-irritation, genetically customized, single nucleotide polymorphisms

## Abstract

Retinol, one of the most powerful cosmetic materials for anti-aging supported by a solid scientific background, exhibits a wide range of type and severity of irritation while showing limited user compliance. The lack of understanding of the mechanism of retinol-induced irritation has been the main hurdle in the development of anti-irritation strategies. Here, we identified 30 genetic markers related to the susceptibility to retinol-induced irritation in the Korean population. Based on the genetic analysis, a novel formula against retinol-induced irritation was developed, which mitigated the molecular pathogenesis—as indicated by the genetic markers—of the retinol-induced irritation. In human tests, this formula effectively decreased retinol-induced irritation. Furthermore, a polygenic risk score model for irritation was constructed and validated. Our comprehensive approach for the analysis of retinol-induced irritation will not only aid the development of anti-irritation strategies to ensure higher user compliance but also contribute to improving the current knowledge about the biological effects of retinoids.

## 1. Introduction

Retinoids, a family of compounds derived from vitamin A that includes both synthetic and natural compounds, have been intensively studied and utilized in diverse biomedical applications such as cancer treatment (acute myeloid leukemia), cervical neoplasia, and skin disorders in the past few decades. The wide application of retinoids, especially retinoic acid, is based on the scientific premise that retinoic acid is involved in numerous biological pathways in nature by triggering interactions between nuclear receptors that control gene expression [1,2,3,4,5].

One of the most striking applications of retinoids is the dermatologic approach, along with pharmaceutical drugs and cosmetic products. Since retinoids were first used for the treatment of acne in the 1940s, its therapeutic efficacy against skin disorders such as actinic keratosis, psoriasis, and ichthyosis is reported [6]. As many scientific studies and clinical studies demonstrated the therapeutic efficacy of tretinoin (all-trans retinoic acid) on photoaging, the development of retinoid derivatives for use as cosmetic ingredients was triggered. Cosmetic retinoids, which include retinyl palmitate, retinyl acetate, retinol, and retinal have been intensively investigated in the commercial market since retinoic acid was prohibited in cosmetic products by global regulations such as Annex II 375 (EC 1223/2009). Some studies have revealed that these cosmetic retinoids are also effective for the treatment of photoaging, whose pathologic characteristics involve fine and coarse wrinkles, skin roughness, and abnormal pigmentation, and even argued that they are potent as tretinoin [7].

However, retinoids cause irritation and some side effects characterized by erythema, scaling, burning, and itching, which are also termed retinoid dermatitis [8]. Some studies have investigated the mechanism of irritation, and it is suggested that diverse mechanisms are involved in retinoid-induced irritation, which include extensive and pervasive inflammation characterized by the release of cytokines and infiltration of immune cells [9], and skin barrier disruption characterized by genetic imbalance of the cornified envelope (CE)-related factors [10,11]. In addition, the phototoxicity of retinoid and its degraded by-products due to its instability under UV and heat has been widely suggested to contribute to the retinoid-induced irritation [12]. However, these observations are insufficient to explain the retinoid-induced irritation thoroughly, and no clear conclusion has been reached to date. For this reason, current retinol-based products usually have a “tedious” anti-inflammatory formula, which is generally and universally used in general commercial products. Therefore, an anti-irritation strategy against retinol based on a strong scientific background has not yet been established.

A genetic approach can be another solution for establishing an anti-irritation strategy against retinol. Genetic variations were extensively studied to discover genes associated with drug efficacy and side effects [13]. Nelson et al. showed that drugs predicted by known genetic associations account for only 2.0% of the drugs at the preclinical stage, although the proportion increases to 8.2% in approved drugs [14]. This implies that focusing on targets discovered by genetic studies would dramatically increase the chance of successful drug development, as demonstrated with drugs for cholesterol or rheumatoid arthritis [15,16,17,18].

Retinol treatment induces changes in the expression of transcription factors related to cellular growth [19] and structural proteins [11]. More specifically, a variety of proteins were implicated in retinol-induced irritation, which include inflammation-related cytokines (*MCP-1, TNF-a*, interferons, and interleukins) [9], retinoic acid receptor (*RARG*) [20], pain-sensing channel transient receptor potential vanilloid 1 (*TRPV1*) [21], and mast cell-activating *GPCR* (MRGPRX2) [22]. However, most genetic studies have been restricted to a single gene or pathway, and the comprehensive mechanism of retinoid dermatitis remains to be elucidated. Therefore, a “candidate gene” analysis of retinoid-induced irritation was conducted to screen for anti-irritants against retinoid dermatitis, and the results of the same will add to the current pool of target genes for studying retinoid dermatitis.

Here, we report a genetically guided anti-irritation strategy using an anti-irritant formula based on the genetic analysis of the Korean population. The results of the genetic analysis undertaken to reveal the genetic factors that govern retinol-induced irritation identified 30 genetic variants of 10 genes including those encoding *EGFR, IL-18, IL-4R, COL6A2*, and *RARB*, to be associated with retinol-induced irritation. We investigated which materials can modulate or alleviate retinol-induced molecular pathogenesis in vitro (*IL-4R*, *COL6A2*, *EGFR*, and *ADIPOQ*). Furthermore, we developed and evaluated a polygenic risk prediction model for retinol-induced irritation in the Korean population. This novel genetics-based approach for the determination of anti-irritation strategies against retinol will contribute to a deeper understanding of retinoids—which is currently lacking—and enable the retinol-based cosmetic product to be more accessible to individuals susceptible to retinol-induced irritation.

## 2. Materials and Methods

### 2.1. Study Design 

**Figure 1:**
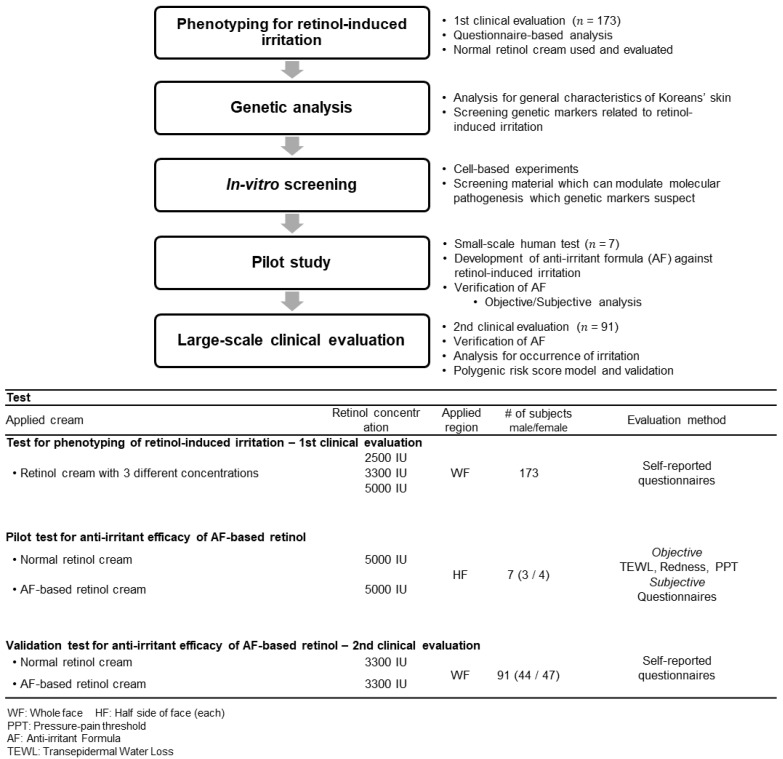
Study design. Upper diagram (flow chart) depicts the overall procedure of the study. The lower table indicates the main three human-based tests. Retinol 1 IU refers to 3 × 10^−5^% *w/w* in the formulation. (2500 IU = 0.075% *w/w*; 3300 IU = 0.1% *w/w*; 5000 IU = 0.15% *w/w*).

This study was designed as shown in Figure 1. Briefly, phenotyping for retinol-induced irritation from the 1st clinical evaluation of 173 Koreans was performed. Through genetic analysis, genetic markers were screened, and anti-irritants and formulas were investigated. Following, a small-scale pilot study (*n* = 7) and large-scale clinical evaluation (2nd, *n* = 91) were performed to verify the anti-irritant efficacy of the newly developed formula.

This study was approved by the Ethics Committee of the LG H&H Institutional Review Board (LGHH-20201217-AA-03, approved on the 17th of December 2020). Prior to participation in the study, human test subjects were informed of possible side effects and consented to participate in the study.

### 2.2. Phenotyping of Retinol-Induced Irritation

In the 1st clinical evaluation, we investigated the irritant property of retinol in 173 Korean individuals by applying increasing concentrations of retinol for 3 days with a resting period of 4 days a week for 3 weeks.

- Using provided retinol cream;
- Providing information through a questionnaire after the experiment;
- Collecting saliva for DNA testing.

Retinol cream was prepared at three different concentrations (level 1, 2500 International Unit (IU), 0.075%; level 2, 3300 IU, 0.01%; level 3, 5000 IU, 0.015%). A corresponding amount of retinol was added to the typical O/W-type cream previously developed in our research institution. The cream formulation is composed of the following components: cetyl stearyl alcohol, glyceryl stearate, PEG-40 stearate, ceteareth-20, beeswax, C14-22 alcohols, C12-20 alkyl glucoside, lecithin, tocopherol, caprylic/capric triglyceride, squalene, cyclopentasiloxane, cyclohexasiloxane, dimethicone/vinyl dimethicone crosspolymer, isocetyl myristate, dipropylene glycol, glycerin, betaine, 1,2-hexanediol, EDTA-3Na, xanthan gum, carbomer, tromethamine, and distilled water. All types of cream used in this study were produced in the LG H&H R&D center by own protocol. The typical cream without retinol was considered as a “non-irritant,” according to patch tests. All participants were informed of the ingredients of the cream prior to the experiment, and the participants who had experienced skin irritation to any of the ingredients in the cream, except for retinol, were excluded from the experiment. The detailed procedure is described in the Supporting Information.

### 2.3. Single Nucleotide Polymorphism (SNP) Microarray

Genomic DNA was extracted from saliva samples using the QIAmp Mini Prep Kit (QIAGEN, Germantown, MD, USA). Genotyping was carried out using the Global Screening assay version 2 chip according to the Illumina Infinium High-Throughput Screening (HTS) assay protocol (Illumina, San Diego, CA, USA). Bead intensity was obtained using the iScan® instrument and subsequently used to yield genotype data using GenomeStudio® software (Illumina, San Diego, CA, USA).

Quality control of the genotype data was preferentially performed under the following conditions: Among the genotyped samples, individuals with a genotype call rate lower than 98% were excluded. For genotyped variants, SNPs with call rates lower than 98%, minor allele frequencies lower than 1%, deviation from Hardy–Weinberg equilibrium (HWE *p*-value < 1 × 10^−6^), and those having more than two alleles were excluded. Among the 173 genotyped individuals, 159 passed the QC criteria and were selected for further analysis.

### 2.4. Assessment of Irritation

The two types of creams, namely the control cream with 5000 IU retinol, and the anti-irritant formula (AF)-based cream with 5000 IU retinol, were administered to seven individuals (three men and four women) in the pilot study. The individuals applied the creams on their face before bedtime in the following manner: one cream on one half of the face and another on the other half of the face. The test subjects were blinded to the cream containing the anti-irritant formula (single-blinded). After three days of application, the subjects were asked to cease the application for 4 days. Skin redness and transepidermal water loss (TEWL) were measured using a chromameter (CR-400, Konica Minolta, Osaka, Japan) and Tewameter^®^ (TM 300 E, Courage + Khazaka electronic GmbH, Köln, Germany), respectively. To measure the pain pressure threshold (PPT), an algometer with a probe diameter of 1 mm combined with a cylinder was manufactured and used. Before the measurement, the faces of the subjects were cleaned and acclimated for 20 min in an air-conditioned room (temperature, 23 ± 2 °C; relative humidity, 50 ± 10%).

Considering that retinoid-induced irritation triggers an extremely wide range of irritation types and severity, it appears that traditional irritation-measuring guidelines mainly focusing on erythema proposed by Frosh and CTFA guidelines [23] do not effectively qualify or quantify these irritations. Therefore, we re-designed the self-evaluation guideline for skin irritation, as shown in Table S1 (see Supplementary Materials). This self-evaluation was performed in a small pilot study and a 2nd large-scale clinical evaluation.

### 2.5. Cell Preparation and In Vitro Experiment

Detailed information regarding cell preparation and in vitro experimental procedures is provided in the Supplementary Information. Briefly, keratinocytes, fibroblasts, mast cells, and TRPV1-overexpressing HEKs were cultured using their own culture protocols and experiments. RT-PCR, β-hexosaminidase release, and calcium influx were analyzed.

### 2.6. Statistical Analysis

Statistical analysis was performed using SNP and Variation Suit (SVS) v8.9.0 (Golden Helix, Bozeman, MT, USA), and PLINK 1.90 (Cambridge, MA, USA) [24]. For candidate gene analysis, SNPs within the candidate genes (including promoter region −2 kb and downstream region 500 bp) were extracted. The statistical significance of the associations and odds ratios were determined by a genotype association test with an additive model. Prior to the calculation of the polygenic risk score, linkage disequilibrium (LD) was examined, and SNPs were selected using Haploview 4.2 (Cambridge, MA, USA). [25]. Alleles that showed an increased tendency for retinol-induced irritation were used for the analysis. The polygenic risk score was calculated as the weighted sum of the odds ratios of each allele [26]. Input data preprocessing, generating a random subset of participants for repeated validation, and calculation of polygenic risk score were conducted using a custom-written code in R v.4.0.3. GraphPad Prism v7.04 (GraphPad Software, San Diego, CA, USA) was used for data visualization. Error bars whose values were derived by dividing each standard deviation by the square root of the number of samples are shown in the figures.

In the analysis of the occurrence rate of irritation based on the dichotomous question (whether retinol cream is irritant: Y/N), the chi-squared test was performed to investigate the statistical significance.

## 3. Results and Discussion

### 3.1. Proposing Target Genes for Screening of Anti-Irritants to Retinol-Induced Irritation

#### 3.1.1. 1st Clinical Evaluation—Topical Application of Retinol and Analysis of Its Irritant Properties

We investigated the irritant properties of retinol and its relationship with skin sensitivity in 173 Korean individuals. They applied retinol for 3 days, which was followed by a rest of 4 days a week for 3 weeks, with each concentration of retinol gradually increasing every week (2500 IU in the 1st week, 3300 IU in the 2nd week, and 5000 IU in the 3rd week). Then, we analyzed the questionnaire received after the experiment to investigate the factors related to retinol-induced irritation.

**Figure 2:**
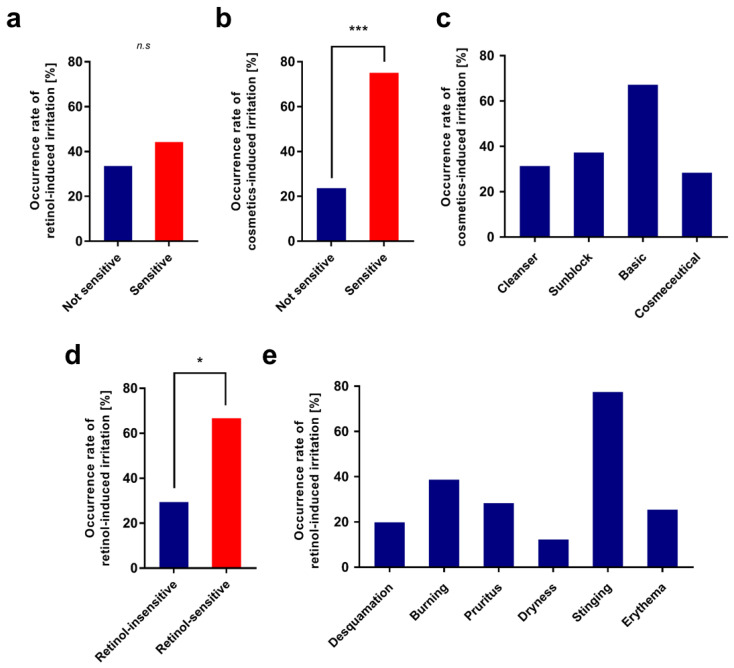
Bar plots depicting the analysis of questionnaires following the test with retinol cream. (**a**) The proportion of individuals who experience irritation to retinol cream according to self-reported skin sensitivity. (**b**) The proportion of individuals who have stopped using cosmetic products due to irritation according to self-reported skin sensitivity. (**c**) The proportion of individuals who experience irritation when using basic cosmetic products. (**d**) The proportion of individuals with a past experience of irritation to retinol-containing products. (**e**) The types of irritation that individuals have experienced during use of retinol-containing topical products; * *p*-value < 0.05; *** *p*-value < 0.001; n.s, not significant.

A higher proportion of participants in the sensitive skin group reported that they experienced irritation compared to the non-sensitive skin group (Figure 2a). It was also shown that individuals who belong to the sensitive skin group were about three times more likely to have past experience of stopping cosmetic product usage due to skin irritation (Figure 2b). Among those with past irritation experiences, most individuals replied that basic cosmetics triggered irritation, which was followed by sunblock, cleanser, and cosmeceutical products (Figure 2c). A higher proportion of individuals who had skin irritation of retinol products in the past answered that they had irritation in this experiment compared to those who had not experienced irritation when using retinol products in the past (Figure 2d). This result revealed that retinol-induced irritation tends to occur repeatedly depending on individuals, which supports the hypothesis that genetic factors might affect the sensitivity to retinol. Earlier studies have shown that genetic variations significantly affect the bioavailability of retinol and retinoid, which support that genetic factors could also govern retinol-induced irritation [27,28].

The type of irritation induced by retinol usage varies greatly among individuals. However, stinging was most common, which comprised about three-fourths of the irritations and was followed by burning, itching, and erythema (Figure 2e).

#### 3.1.2. Candidate Gene Analysis for Retinol-Induced Irritation

To discover genetic variants associated with skin sensitivity to retinol, we chose 14 candidate genes that are well-known for their functions in retinoid metabolism and skin sensitivity (Table S2). We referred to previous transcriptomic analysis of the 3D reconstituted skin under treatment of sensitizers [29].

A total of 319 SNPs located within the candidate genes were selected and used for the association analysis using the additive model. Thirty SNPs were significantly associated with retinol-induced irritation, none of which have been previously reported (*p* < 0.05; Table 1). In detail, a total of 12 SNPs were found in *RARB*, three in *EGFR*, three in *CD44*, two in *IL18*, two in *IL4R*, and four in *BCL2*. The other four genes, *CD86*, *RXRB*, *MMP10*, and *COL6A2*, included single SNPs. The discovered SNPs belonged to 10 genes, two of which were retinol-related genes, and the remaining eight were related to general skin sensitivity according to earlier studies. Among the 10 genes, we selected the most important three genes in terms of skin irritation: *COL6A2, EGFR*, and *IL-4R*.

*COL6A2*, which encodes one of the three alpha chains of type VI collagen found in most connective tissues, has been shown to regulate dermal matrix assembly and fibroblast motility [30], and it contributes to tissue remodeling and wound healing [31,32]. This defect leads to keloid formation [33] and abnormal skin phenotypes [34]. Epidermal growth factor receptor (EGFR) is an important regulator of epidermal barrier function. EGFR signaling has been shown to inhibit the competence of the cornified envelope and disrupt tight junction barrier function in epidermal keratinocytes [35,36]. In addition to EGFR signaling, another wide range of factors such as ADAM17 (a disintegrin and metalloproteinase 17) [37] and TRP channel [38] are also believed to affect the epidermal barrier function systematically associated with EGFR.

In the past few decades, the functions and roles of interleukin 4 (IL-4) and its receptor, IL-4R, have been extensively investigated. As a key regulator in the humoral and adaptive immune system, IL-4, which is primarily secreted from mast cells and Th2 cells, induces the differentiation of Th2 cells and stimulates B cells. Although its role is not clearly understood, many studies have pointed out that IL-4 may drive extensive pro- and inflammation processes, while its defect was shown to drive allergic disease, Alzheimer’s disease, and tumors [39]. Along the same axis for the IL-4-related immune response, IL-4R is ubiquitously expressed on various immune cells in both the innate and adaptive immune systems. IL-4R is a common receptor for both IL-4 and IL-13 [40]. Unlike IL-4, which directly affects Th2 differentiation, IL-13 is also responsible for activating mast cells, which control eosinophil function, and it has immunosuppressive and anti-inflammatory effects on macrophages by suppressing pro-inflammatory cytokines and chemokines [41].

### 3.2. Top–Down Approach: Screening Anti-Irritants In Vitro 

#### 3.2.1. Tissue Repair-Related Genes, COL6A2, and EGFR 

Based on the selected genes, we screened for anti-irritants that could modulate molecular pathogenesis, such as controlling the expression of the target genes.

Earlier reports revealed that retinoids induce physiological and morphological changes in the skin barrier [8,42,43]. It was observed that the treatment of skin tissue with retinoic acid ex vivo and in vivo elevates transepidermal water loss (TEWL), and several studies have revealed that this disruption of skin barrier function is mediated by an imbalanced expression pattern of the cornified envelope and tight junction-related genes. The downregulation of filaggrin (*FLG*), loricrin, and *CLDN1*, and upregulation of *CLDN2*, *CLDN4*, and a significantly different expression of serpin family member genes were observed in an earlier study, which is thought to be the main cause of retinoid-induced irritation [10,11]. 

**Figure 3:**
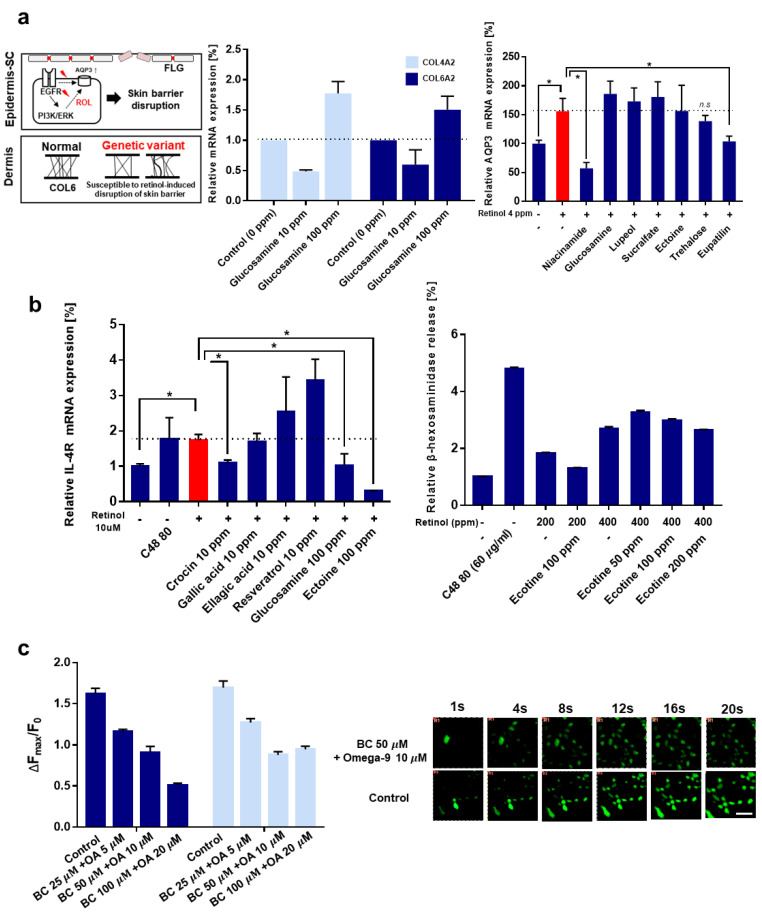
The analysis for mRNA expression to screen anti-irritants that could modulate irritation-associated molecular pathogenesis. (**a**) Investigation for skin barrier disruption-associated molecular pathogenesis. Schematic diagram for the approach for genetic modulation for retinol (ROL)-induced skin barrier disruption. (left) The relative mRNA expression of COL4A2 and COL6A2 when fibroblasts were treated with glucosamine (middle) and relative mRNA expression of AQP3 (keratinocyte, right). (**b**) Investigation for inflammation (mast cell driven)-associated molecular pathogenesis. Relative mRNA expression of IL-4R when RBL-2H3 was treated with 10 µM retinol and various candidates. (**c**) Neurogenic inflammation mediated with TRPV1 induced by retinol and antagonistic effect by 4-t-butylcyclohexanol and omega-9; Three conditions for combination: (1) 4-t-butylcyclohexanol 25 μM and omega-9 5 μM, (2) 4-t-butylcyclohexanol 50 μM and omega-9 10 μM, and (3) 4-t-butylcyclohexanol 100 μM and omega-9 20 μM, BC, 4-t-butylcyclohexanol; OA, omega-9 oleic acid; A 50 µm scale bar (white) was shown; * *p* < 0.05; n.s, not significant; error bar was shown; ppm, parts per million.

We hypothesized that individuals with genetic variations in *COL6A2* and *EGFR* tend to be susceptible to the weakening of skin barrier function by retinol, which leads to retinol-induced irritation (Figure 3a). We also considered that apart from collagen VI, collagen IV is also an important factor in the epidermal basement membrane and wound-healing process, although its exact role in the skin tissue remains unclear [44]. We hypothesized that a deficiency or abnormality of collagen VI and IV leads to susceptibility to retinol-induced irritation, and the overexpression of both types of collagen could attenuate the retinol-induced weakening of the skin barrier. Among the various substances, we observed that glucosamine increased the expression of COL6A2 and COL4A2 by 1.5-fold and 1.7-fold, respectively (Figure 3a, middle panel). The modulatory role of glucosamine on COL6A2 and COL4A2 has not been reported before, which implies that these observations are scientifically important.

In the case of EGFR, we carefully considered previous research indicating that retinoic acid induces the overexpression of aquaporin 3 (*AQP3*) through the *EGFR/ERK* pathway in human keratinocytes [45]. In the first report, we observed that not only retinoic acid but also retinol induced the *AQP3* expression by approximately two-fold in fibroblasts (Figure 3a right panel). The attenuation effect of *AQP3* overexpression by niacinamide (nicotinamide) is similar to that found in an earlier study. Among the various substances, trehalose and eupatilin attenuated retinol-induced *AQP3* overexpression. Eupatilin (5,7-dihydroxy-3’,4’,6-trimethoxyflavone), a type of flavonoid found in *Artemisa asiatica*, was reported to enhance the skin barrier function under pathological conditions in vivo [46].

Additionally, we investigated the expression of FLG, which is a prime indicator of skin barrier function. Similar to previous in vivo studies [47], we observed that retinol significantly decreased the expression of FLG by approximately 45%, and this decrease was reversed by niacinamide, glucosamine, and sucralfate (Figure S1a, see Supplementary Materials).

Collectively, glucosamine, trehalose, and sucralfate could alleviate retinol-induced skin barrier disruption by modulating the expression of *COL6A2, AQP3*, and *FLG*.

#### 3.2.2. Inflammatory Gene: *IL-4R*

Then, we tried to identify anti-irritants related to inflammatory genes. Based on our results of genetic analysis, it has been shown that individuals who are prone to retinol-induced irritation tend to have SNP markers for *IL-4R* but not *IL-4*. First, we investigated whether treatment with retinol modulates the expression of *IL-4* or *IL-4R* in mast cells, which is also a primary driver of *IL-4*-related inflammation. Retinol induced the overexpression of *IL-4R* 1.74-fold. Although retinol did not induce the overexpression of *IL-4* (Figure S1b, see Supplementary Materials), it was observed that ectoine effectively decreases the expression of *IL-4* of the mast cell, which is coincident with earlier studies that argued the anti-inflammatory effects of ectoine in some disease models such as IBD (inflammatory bowel disease) and allergic airway disease [48,49]. More specifically, earlier studies showed that ectoine could normalize the expression of *IL-4* in the CNP (carbon nanoparticle)-induced lung inflammation in an in vivo model, supporting our experimental results [50].

Crocin, glucosamine, and ectoine alleviated the retinol-provoked overexpression of *IL-4R* in mast cells, with a decrease of 37.03%, 41.97%, and 82.59%, respectively. Ectoine (1,4,5,6-tetrahydro-2-methyl-4-pyrimidinecarboxylic acid), a natural compound found in several halophilic bacteria, was shown to modulate many cytokines and chemokines under inflammatory conditions, although the role of ectoine in ameliorating the expression of *IL-4R* has not yet been elucidated. Our observations offer novel insights into the function of ectoine. Previous study by our group showed that ectoin at an optimum concentration of 100 ppm showed the highest anti-inflammatory effect against the effects of retinoids and negligible cytotoxicity in the cell-based in vitro experiment (data not shown).

We also measured the β-hexosaminidase release rate of mast cells, which is an indicator of mast cell degranulation (Figure 3b, right panel). Ectoine did not reduce retinol-induced β-hexosaminidase release in the 400 ppm retinol, although it reduced retinol-induced β-hexosaminidase release in the 200 ppm retinol.

Collectively, it can be concluded that ectoine can ameliorate *IL-4* and *IL-4R*–related inflammation induced by retinol. Considering the previous research that ectoine intervenes in the activation of *EGFR* in CNP-induced lung inflammation [50], ectoine also appears to be beneficial for skin barrier disruption induced by retinol.

#### 3.2.3. Neurogenic Inflammation, Adiponectin, and TRPV1

One distinguishing feature of retinol-induced irritation in extremely retinoid-sensitive patients is allergy-like reactions such as rapid burning and sting sensation, itchiness, rapid diffusive edema, and rash. A study revealed that retinoids, including retinol and retinoic acid, activate *TRPV1* [21]. Therefore, we hypothesize that retinol-induced irritation, especially promptly occurring within a few minutes, is mediated by the activation of *TRPV1* by retinol.

*TRPV1*, a nonselective cation activated by various physicochemical stimuli, has been widely studied, especially its role in the skin. [51,52,53,54] Although *TRPV1* did not emerge in the previous genetic analysis, it should be noted that adiponectin (*ADIPQ*) appeared in some analysis models with low to moderate statistical significance. An earlier study of the Korean population showed that the “sensitive” skin phenotype appears to be related to adiponectin deficiency, which consequently contributes to the upregulation of TRPV1 [55].

*TRPV1*-overexpressing HEK293 cells were treated with retinol, and the activation of *TRPV1* was confirmed by imaging calcium influx. We observed that retinol could activate *TRPV1* in a dose-dependent manner (Figure S1c, see Supplementary Materials). At concentrations above 50 μM, the agonistic effect on *TRPV1* seemed to reach a plateau. Based on a previous study, we investigated whether 4-t-butylcyclohexanol and omega-9 oleic acid can antagonize *TRPV1* activation induced by retinol. As expected, both substances can antagonize *TRPV1*, while at low dosage, omega-9 oleic acid was more potent than 4-t-butylcyclohexanol (Figure S1d, see Supplementary Materials). The combination of these two materials showed a slight synergistic effect on the inhibition of *TRPV1* by retinol. Three different concentrations of the combination of 4-t-butylcyclohexanol and omega-9 showed reduced activation of *TRPV1* induced by retinol, 28.25%, 43.73%, and 68.50%, respectively. Calcium influx into cells mediated by the activation of *TRPV1* was observed under fluorescent microscopy (Figure 3c).

In conclusion, we verified that retinol-induced irritation could also be related to neurogenic inflammation mediated by *TRPV1* activation in an in vitro model, and 4-t-butylcyclohexanol and omega-9 oleic acid could be utilized to mitigate neurogenic inflammation by antagonizing *TRPV1*.

### 3.3. Anti-Irritation Efficacy of the Formula; Human Tests

Based on anti-irritant substances verified from the in vitro experiment guided by genetic analysis, we prepared the AF (Anti-irritant Formula) against retinol-induced irritation, which consisted of glucosamine 0.1%, trehalose 2%, ectoine 2%, sucralfate 0.1%, omega-9 1%, and 4-t-butylcyclohexanol 0.7% with retinol. (standardized as an active concentration)

**Figure 4:**
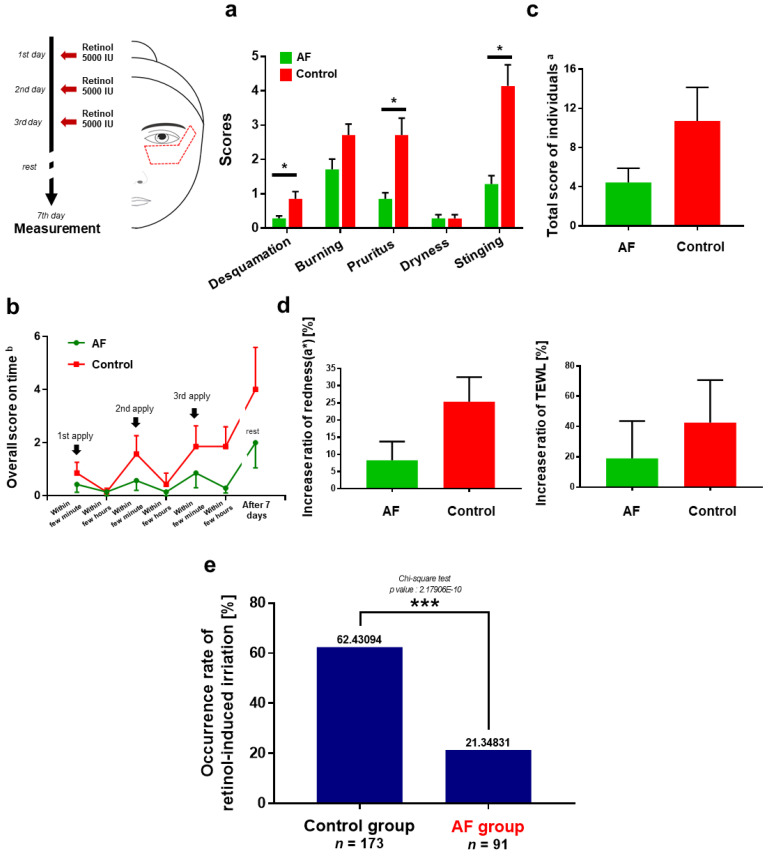
Schematic and plots depicting the anti-irritant efficacy of AF. Panel (**a**–**d**) indicate the results of the small pilot study (*n* = 7). Left schematic diagram shows the test schedule and procedure and test area (red dotted) results of the face that was evaluated for the measurement of TEWL and skin redness. (**a**) Average of the irritation scores that the test subjects experienced during the test period. (**b**) Overall irritation score on each time point. Over irritation score ^b^ refers to the sum of scores of all types of irritation experienced by the individuals. (**c**) Total irritation score of the individuals. Total scores ^a^ for each type of irritation experienced by the individual during the test period were averaged. Overall scores ^b^ on each time point were summed. (**d**) Skin redness and transepidermal water loss (TEWL) measured by chromameter and tewameter. The increased ratios (%) for individuals were averaged. (**e**) Occurrence rate of retinol-induced irritation. Test human subjects were asked a dichotomous question, whether “retinol-based cream is irritant or not”, based on their own subjective criteria. A chi-square test was performed to investigate statistical significance. (*p*-value: 2.17906E-10); * *p*-value < 0.05; *** *p*-value< 0.001; AF, Anti-irritant Formula against retinol-induced irritation; error bars are shown.

First, we performed a pilot study with seven individuals to quantitatively verify the effectiveness of the AF. When the irritation score of each type of irritation for the individual was summed during the test period, it was shown that compared to the control retinol cream, AF effectively decreased retinol-induced irritation, especially desquamation (66.67% decrease), burning (68.42%), and stinging (68.97%), and this amelioration of symptoms was statistically significant (Figure 4a). Interestingly, the test subjects claimed that they did not experience dryness during the test period, although they had undergone significant desquamation and dryness after 10 days, even over 2 weeks. This observation is consistent with a previous study that claimed that retinoic acid induces skin dryness after 9 days of treatment and persists until 18 days by showing a reduced turnover rate of the stratum corneum (15.8 days) compared with the placebo (18 days) [56].

Next, the overall score of irritation over time was investigated (Figure 4b). As may be expected, the irritation was observed within a few minutes after applying the retinol cream and decreased after a few hours. We found that our AF effectively reduced irritation, especially after the third application of cream. While the level of irritation increased even after ceasing application of retinol cream, the anti-irritation effect of AF remained and alleviated the retinol-induced irritation post-procedure. Collectively, AF reduced the total irritation score of individuals during the test period by 58.3% (Figure 4c).

At the 7th day, we measured the TEWL and redness (Figure 4d). AF reduced both the increase in TEWL and the redness induced by retinol. The average increase in redness (a*) was 8.31% for AF and 8.31% for control. Skin redness, a clinical feature of cutaneous vasodilation, is an indicator of inflammation [57]. Previous studies have shown that retinoids induce cutaneous inflammation mediated by the release of *MCP-1* and *IL-8* from fibroblasts. It is noteworthy that as shown in the previous section, retinol activates macrophages (overexpression of *IL-4R*), which is the prime driver of cutaneous vasodilation [9].

In the TEWL analysis, the retinol cream based on AF increased TEWL by 19.07% for test subjects individually, while the control retinol cream increased TEWL by 42.54%. This clinical observation is similar to that of a previous report indicating that retinoids temporarily induce an increase in TEWL in vivo in mice and humans [42,58]. Although the statistical significance was low, the mitigation effect of AF on the increase in TEWL and redness may be expected.

We also measured the pressure–pain threshold using an algometer with a tip with a radius of 1 mm (Figure S2, see Supplementary Materials). The sensitive skin of psoriasis patients was reported to show a decreased pressure–pain threshold compared to normal individuals [59]. The differences in threshold values of the individuals before and after treatment with retinol cream were averaged. It was observed that retinol decreased the pressure–pain threshold (PPT), and AF increased the decreased PPT significantly compared to the control. It seems to be mediated by the anti-inflammatory and antagonistic effects of AF on *TRPV1*. Earlier studies have shown that neurogenic inflammation is highly related to the mechanical sensitization of cutaneous nociceptors [60], and mice lacking functional *TRPV1* display attenuated mechanical hyperalgesia to noxious mechanical stimuli [61]. One human clinical study also demonstrated that a *TRPV1* antagonist (V116517) significantly increased the PPT of the skin under various conditions [62].

To verify the anti-irritant effect of AF in the independent group, we grouped together all the human subjects, which consisted of 44 males and 47 females with ages ranging from 27 to 50 years. The test subjects were asked a dichotomous question, whether “retinol-based cream is irritant or not”, based on their own subjective criteria. This response ratio was compared to that of the first large-scale clinical evaluation of 173 individuals who had used a normal retinol cream in order to derive the anti-irritant effect of AF.

The occurrence rate of retinol-induced irritation was calculated and compared (Figure 4e). A 64.43% occurrence rate was observed in the group that had used normal retinol cream, whereas an occurrence rate of only 21.35% was observed in the group that had used AF-retinol cream, which indicated a decrease of 65.80%.

### 3.4. Polygenic Risk Prediction Model for Retinol-Induced Irritation

As anti-irritants in AF were screened from the associated genes, we constructed a polygenic risk prediction model to evaluate the cumulative genetic effect of multiple loci on retinol-induced irritation. A polygenic risk prediction model was constructed using 26 significant SNPs selected after LD analysis based on |D’| and r^2^ values(coefficients for degree to which an allele of one SNP is inherited or correlated to the allele of another SNP) between SNPs. Then, the risk scores of all 159 samples were visualized.

The test subjects of the second large-scale clinical test were arbitrarily categorized into three groups: high (>75, 31 individuals), middle (65–75, 33 individuals), and low risk score (<65, 27 individuals) (Figure S3, see Supplementary Materials). As characteristics of retinol-induced irritation in each group, the high-risk score group exhibited dryness and desquamation after the last third treatment of retinol compared to the other groups (Figure S4, see Supplementary Materials). This result supports our hypothesis that skin barrier disruption with increased TEWL plays an important role in retinoid-induced irritation, as SLS exhibited similar clinical features and anatomical changes [63].

**Figure 5:**
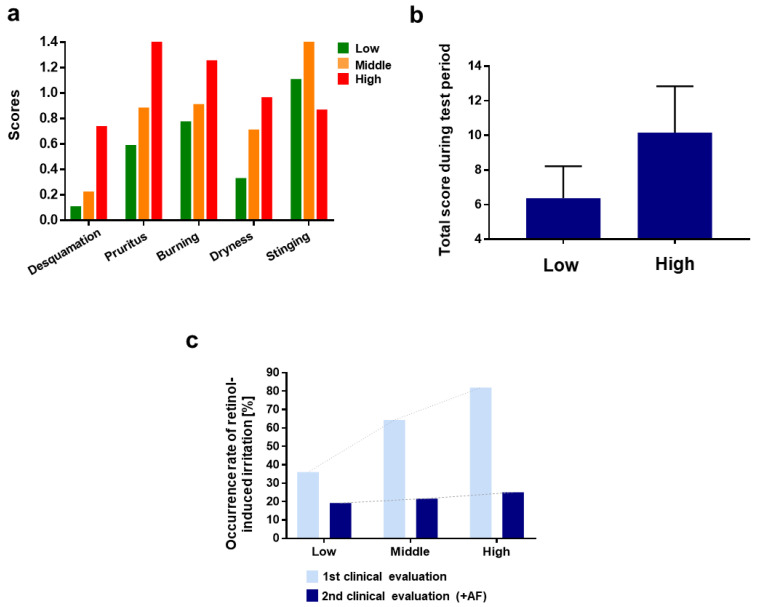
Bar plots illustrating polygenic risk score model and validation. 3300 IU retinol cream with AF to 91 individuals. Test subjects were categorized into three groups: high-risk score (>75, 31 individuals), middle-risk score (65~75, 33 individuals), and low-risk score (<65, 27 individuals) (**a**) Irritation score of each type of irritation for three risk score groups. The scores for types of irritation of individuals during the test period were summed and averaged by risk score groups. (**b**) Total score during test period. (**c**) Comparison of occurrence rate of irritation on each risk-score group. The dichotomous question (whether retinol cream is irritant: Y/N was asked to test subjects. In the first clinical evaluation, the retinol without AF was given. The retinol with AF was given in second clinical evaluation.

As shown in Figure 5a, the group with a high-risk score consisted of the most irritation-experienced participants among the other groups. “Pruritus” was the most dominant type of irritations for the high-risk score group compared to the other groups. The participants in the high-risk score group experienced pruritus most frequently on the 1st day of retinol treatment, and the degree of pruritus decreased gradually over time. The high-risk score group seemed to experience allergy-like symptoms related to histamine and mast cell-mediated systems (Figure S4, see Supplementary Materials).

The total score during the test period was calculated as 6.370 and 10.161 for the low-, middle-, and high-risk score groups, respectively (Figure 5b).

The efficacy of AF, according to the polygenic risk score model, was investigated. The results demonstrated that AF alleviated retinol-induced irritation, and the efficacy could be predicted using the polygenic risk score model (Figure 5c).

Consequently, these experimental results and observations not only demonstrate the anti-irritation effect of AF but also imply that proposing an appropriate dosage of retinol by estimating the susceptibility to retinol-induced irritation based on individual genetic information is applicable, which can finally offer genetically the most-optimized usage protocol to patients with higher compliance.

### 3.5. Further Consideration; Acute vs. Chronic Irritation?

As mentioned before, allergy-like reactions, which including rapid burning and stinging sensation, itchiness, and rapid diffusive edema and rash were observed in retinol-sensitive individuals. These reactions, which occurred within a few minutes, sometimes even a few seconds, cannot be clearly explained by previous retinoid-mechanistic studies that have mainly focused on cytokine production by keratinocytes or fibroblasts [9], or the immune system in which T cells, B cells, or complementary activation are mainly involved. Although it is undeniable that skin barrier disruption is the main cause of irritation, it does not fully explain the acute irritation that promptly occurs.

In the first clinical evaluation, it was observed that about 5% of the test subjects experienced violent and severe above-mentioned reactions and were dropped from the test. However, detailed investigations have not been performed regarding the intolerance of retinoid depending on race, sex, or age, and it has been reported that the Asian population is more prone to these reactions, which urges the necessity for the consideration of rapid-provoked irritation for the further development of retinoid-based products. Our human test results suggest that neurogenic inflammation, or a similar prompt mechanism, is also involved in retinol-induced irritation. The burning sensation and stinging were dominantly observed at each retinol treatment within a few minutes in all three groups and repressed over time.

Here, these are some points that deserve consideration.

1. As reported earlier, retinol may be converted into retinaldehyde (retinal) and retinoic acid to possess the bioavailability at which *RAR* or *RXR* binding is involved.
2. However, the homeostasis of retinoids in the skin is very tightly controlled [64], and paradoxically, it has not been experimentally proven that retinol converts into retinoic acid in vitro and ex vivo [65,66].
3. Under retinol treatment, fibroblasts or keratinocytes activate the circumjacent cutaneous immune system as quickly as within a few minutes by producing *IL-1* or *IL-6* through the interaction between *RAR* and retinoic acid, to which the retinol should be converted.

Based on these considerations, it seems more reasonable that acute irritation within a few minutes may be driven by the histamine–mast cell system or neurogenic inflammation promptly, which subsequently triggers extensive and pervasive inflammation, leading to long-term irritation.

## 4. Conclusions

Although retinol is regarded as one of the most effective and attractive cosmetic materials based on its strong scientific background, its characteristic irritation has remained a major hurdle for the ubiquitous anti-aging strategy, since only a handful of individuals are allowed to utilize it. Some efforts to reveal the mechanism of retinoid-induced irritation were attempted, although the in-depth mechanism remains unclear. The lack of understanding of retinol-induced irritation regarding why the types and degree of irritation vary according to ethnicity, sex, or even one person to the next has delayed the development of anti-irritation strategies. Here, we identified genetic markers related to retinol-induced irritation in the Korean population and developed a novel formula for anti-irritation against retinol. As a genetically guided top–down approach, the series of in vitro experiments revealed that glucosamine, sucralfate, trehalose, ectoine, 4-t-butylcyclohexanol, and omega-9 fatty acid could mitigate the irritation-associated molecular pathogenesis for *COL6A2*, *EGFR*, and *IL-4R*. Our developed formula against retinol-induced irritation decreased skin redness and TEWL in the objective analysis, and it also decreased the individuals’ degree of irritation on a subjective analysis in both small-scale pilot studies and large-scale human tests. An anti-irritant strategy was developed, and a polygenic risk score model for the prediction of individual irritation was developed.

Here, our strategies against retinol-induced irritation include (1) disclosing and screening genetic markers related to retinol-induced irritation, (2) a formula for reducing irritation based on in vitro verification of whether this formula can modulate molecular pathogenesis suspected by genetic markers, and (3) a polygenic risk score model for the prediction of irritation. Our approach will improve the compliance of patients who require retinol for various purposes in the future, while suggesting significant scientific clues to basic retinoid science, which remains to be elucidated.

## Figures and Tables

**Table 1:** SNPs significantly associated with retinol-induced irritation.

SNP	Gene	Chr:Position	Allele ^1^	Position	MAF	Effect Size ^2^	*p*-Value
rs6550923	*RARB*	3:24924495	A > G	Intron	0.368	0.52	0.0267
rs59964722	*RARB*	3:24950518	A > G	Intron	0.108	0.27	0.0109
rs10510553	*RARB*	3:24966768	A > G	Intron	0.221	2.27	0.0159
rs4280597	*RARB*	3:25053482	A > G	Intron	0.255	0.51	0.0315
rs1021701	*RARB*	3:25058757	A > G	Intron	0.255	0.51	0.0315
rs1604003	*RARB*	3:25068617	C > A	Intron	0.123	0.36	0.0213
rs6804842	*RARB*	3:25106437	G > A	Intron	0.304	2.15	0.0180
rs321526	*RARB*	3:25240188	C > A	Intron	0.245	2.08	0.0378
rs73042351	*RARB*	3:25260568	A > G	Intron	0.044	4.64	0.0420
rs73151296	*RARB*	3:25305813	A > G	Intron	0.059	4.11	0.0302
rs1286733	*RARB*	3:25613372	A > G	Intron	0.289	0.44	0.0107
rs3773439	*RARB*	3:25617775	A > G	Intron	0.382	0.46	0.0089
rs1129055	*CD86*	3:121838319	A > G	Exon	0.373	1.79	0.0452
rs3117040	*RXRB*	6:33164735	A > C	Intron	0.039	0.15	0.0344
rs6970262	*EGFR*	7:55259763	G > A	Intron	0.103	2.91	0.0330
rs2740762	*EGFR*	7:55261342	C > A	Intron	0.064	4.68	0.0152
rs2293348	*EGFR*	7:55266757	G > A	Intron	0.083	0.20	0.0073
rs996076	*CD44*	11:35210798	A > G	Intron	0.123	2.43	0.0480
rs2295756	*CD44*	11:35241229	G > A	Intron	0.353	1.77	0.0499
rs10128586	*CD44*	11:35245907	A > G	Intron	0.221	0.50	0.0490
rs17099562	*MMP10*	11:102648527	G > A	Intron	0.177	0.42	0.0290
rs5744247	*IL18*	11:112026156	G > C	Intron	0.348	0.49	0.0136
rs187238	*IL18*	11: 112034988	C > G	Intergenic	0.123	2.43	0.0480
rs1110470	*IL4R*	16:27336427	G > A	Intron	0.275	2.32	0.0039
rs3024530	*IL4R*	16:27350687	G > A	Intron	0.417	2.34	0.0028
rs12454712	*BCL2*	18:60845884	A > G	Intron	0.480	0.58	0.0362
rs8098848	*BCL2*	18:60857793	A > G	Intron	0.446	0.45	0.0086
rs2062010	*BCL2*	18:60873082	G > A	Intron	0.431	0.53	0.0308
rs17841945	*BCL2*	18:60881555	G > A	Intron	0.078	3.06	0.0464
rs117668143	*COL6A2*	21:47551909	G > A	Exon	0.049	12.79	0.0020

^1^ The alleles were described as ‘major allele > minor allele’. ^2^ Effect size (odds ratio) of minor allele. MAF, minor allele frequency; SNP, single nucleotide polymorphism; Chr, chromosome.

## Data Availability

The data that support the findings of this study are available from the corresponding author upon request.

## Supplementary Materials

The following are available online at https://www.mdpi.com/article/10.3390/pharmaceutics13122006/s1, Experimental procedure during the first clinical evaluation in vitro experimental procedure. Table S1: Self-evaluation index for retinol-induced irritation. To investigate the diverse type of retinol-induced irritation, the new scoring index was developed. Table S2: List of candidate genes used in the genetic analysis and known functions related to skin sensitivity. Figure S1: In-vitro experiment to screen anti-irritants which could modulate irritation-associated molecular pathogenesis. (a) Investigation for skin barrier disruption-associated molecular pathogenesis. Relative mRNA expression of FLG. Keratinocyte HaCat was experimented. Retinol 4ppm was treated. (b) Investigation for inflammation (mast cell driven)-associated molecular pathogenesis. relative mRNA expression of IL-4 when RBL-2H3 was treated with 10μM retinol and various candidates. (c) Neurogenic inflammation mediated with TRPV1 induced by retinol and (d,e) antagonistic effect by 4-t-butylcyclohexanol(BC) and omega-9(OA). Figure S2: Pressure pain threshold (PPT) measured algometer whose probe has the diameter of 1mm. Control refers to retinol-treated area without AF. Negative control refers to non-treated area. Figure S3: Histogram for risk score on test individuals. 1st clinical evaluation with the aim to investigate to disclose the genetic marker which is regarded to retinol-induced irritation. The 173 people were tested and analyzed. Retinol cream without AF were given. (Left panel) 2nd clinical evaluation with the aim to validate the prediction model for retinol-induced irritation. Retinol cream with AF were given. The test subjects were divided into three groups with following criteria; Low (≤65), Middle (65~75), High (75<) (Right panel). Figure S4: Patterns of irritation in each risk score group. Each type of irritation was averaged.
