# Supplementary material for: Anti-Irritant Strategy against Retinol Based on the Genetic Analysis of Korean Population: A Genetically Guided Top–Down Approach
Source: Pharmaceutics. 2021 Nov 25;13(12):2006. doi: 10.3390/pharmaceutics13122006 (PMC8706521; doi:10.3390/pharmaceutics13122006)
Supplement: Supplementary file 1 [file pharmaceutics-13-02006-s001.zip › SM-1398811/pharmaceutics-1398811-supplementary-update.pdf]

pharmaceutics

Supplementary Materials: Anti-Irritant Strategy against Retinol Based on the Genetic Analysis of Korean Population: A Genetically Guided Top–Down Approach

Seongsu Kang, Kyunghoe Kim, Seung-Hyun Jun, Seonju Lee, Juhyun Kim, Joong-Gon Shin, Yunkwan Kim, Mina Kim, Sun-Gyoo Park and Nae-Gyu Kang

1. Experimental procedure in 1st clinical evaluation for phenotyping

We recruited 173 Korean people for the testing of irritant property of retinol cream for individuals. We analyzed questionnaire received after the experiment to investigate what kind of factors are related to retinol-induced irritation. The participants were asked to topically apply a small amount (about 0.3 g) of the provided cream on their faces. In the first week, the lowest concentration (2,500 IU) was used for three consecutive days, and four days were given for rest before moving on to use the second concentration, and so on. Through the questionnaire, the participants answered questions regarding 1) general skin sensitivity, 2) experience of quitting cosmetics due to irritation, 3) category of cosmetic products that they are sensitive to, 4) irritation for retinol-based products in the past, 5) time point when the irritation emerged during the experiment, and 6) the type of irritation they experienced during the experiment. Participants who answered their skin is 'very sensitive' or 'sensitive' were regarded as having sensitive skin, and who answered their skin is 'somewhat sensitive' or 'not sensitive' were regarded as having non-sensitive skin, as previously described [1–3]. We divided the participants into "case group" and control group for retinol-induced irritation. Case group consisted of the people who experienced their first irritation within two weeks, and during the use of provided retinol cream. Control group was defined by the people who did not experience irritation at all, or until they finished the use of the cream with the highest retinol concentration (5,000 IU).

2. In-vitro experimental procedure

2.1. Cell culture and preparation

Fibroblasts were cultured with Dulbecco's modified Eagle's medium (DMEM) (Gibco, Grand Island, NY, USA) supplemented with 10% FBS (Gibco), penicillin-streptomycin (Gibco) at 37 °C with 5% CO_2. HaCaT were cultured with DMEM supplemented with 10% FBS, penicillin-streptomycin (Gibco), 1 mM Sodium Pyruvate (Gibco), 2 mM L-glutamine (Gibco), and 0.01 mM $CaCl_2$ (Sigma Aldrich, USA) at 37 °C with 5% CO_2.

The rat mast cell line RBL-2H3 was obtained from the American Type Culture Collection (ATCC, Rockville, MD, USA). The cells were maintained in DMEM supplemented with 10% (*v*/*v*) heat-inactivated fetal bovine serum (Gibco) and 100 units/mL penicillin-streptomycin (Gibco) at 37 °C in a humidified 5% CO_2 atmosphere. Cells were detached with trypsin-EDTA solution (Gibco), washed with phosphate buffered saline (PBS, pH 7.2), and resuspended in fresh medium for use in subsequent experiments.

TRPV1-overexpressing transgenic cell line whose base cell line is HEK293 was manufactured by Creative Biogene (USA). The Relative mRNA expression level of transgene TRPV1 was 1082.637-fold compared to normal HEK293. The cell was cultured in high glucose DMEM containing 10% FBS, and puromicin which is for antibiotics and selection for transgenic cell line. The cell was maintained under the confluence of 80%.

2.2. RT-qPCR

Fibroblasts and HaCaT were seeding in 6-well plates and incubated for 24 h at 37 °C. In the case of HaCaT, retinol was treated for an additional 24 h in serum-free fresh medium before substances treatment. After incubation, the substances at various concentrations were introduced in serum-free fresh medium. Following incubation for 24 h at 37 °C, RNA was extracted from Fibroblast and HaCaT using RNeasy mini kit (Qiagen, Germany). Total RNA (1 ug) was reverse-transcribed into cDNA using cDNA synthesis kit (Philekorea, Korea) following manufacturer's protocol through Veriti 96 Well Thermal Cycler (Applied Biosystems, USA). The following temperature protocol was used for reverse transcription: 42 °C for 30 min and 72 °C for 10 min. Amplification of cDNA was performed using the StepOnePlusTM RT-PCR system (Applied biosystems) using TaqMan™ Universal PCR Master Mix (Applied Biosystems) according to protocol by manufacturer. The following thermocycling conditions were used for qPCR: 30 cycles at 95 °C for 45 sec, 60 °C for 1 min and 72 °C for 45 sec. qPCR was performed using the following commercial Taqman primers: COL4A1 (Hs00266237_m1); COL6A1 (Hs01095585_m1); AQP3 (Hs01105469_g1); FLG (Hs00856927_g1) Experimental results were calculated by 2-ΔΔCt formula and presented as relative values for housekeeping gene, GAPDH.

For the expression of mast cell, the RBL-2H3 cells (6×10^5 cells/well) were seeded into 6-well plates and incubated overnight. The cells were washed twice with serum-free medium and were preincubated for 4 h with test agents in serum-free medium at the indicated concentrations. They were then treated with 10 μM Retinol or 60 μg/ml C48/80 (Sigma-Aldrich, St. Louis, MO, USA), and incubated for 24 h at 37 °C with 5% CO2. Total RNA was prepared using Qiagen RNeasy kit according to the manufacturer's instructions as described above. The total RNA (1 μg) was used as the template for cDNA synthesis and PCR using the Power SYBR® Green PCR Master mix (Applied Biosystems, foster City, CA, USA). The following primers were used: GAPDH sense 5′- ACCACAGTCCATGCCATCAC -3′; GAPDH antisense 5′-TCCACCACCCTGTTGCTGTA-3′; IL-4 sense 5′- ACCTTGCTGTCACCCTGTTC-3′; IL-4 antisense 5′-TTGTGAGCGTGGACTCATTC-3′; IL-4R alpha sense 5′ –ATCTGCGTGCTTGCTGGTTCT-3′; IL-4R alpha antisense 5′-CTGGTATCTGTCTGATTGGACCG-3′.

2.3.β-. hexosaminidase release assay

The inhibitory activity of test agents against the release of β-hexosaminidase from RBL-2H3 cells was evaluated according to Yi Zheng et al. with slight modifications.[4] RBL-2H3 cells in 24-well plates were incubated overnight at 37 °C with 5% CO2. The cells were washed twice with serum-free medium and were preincubated for 4 h with test agents in serum-free medium at the indicated concentrations. They were then treated with retinol or 60 μg/ml C48/80 for 30 min to evoke pseudo-allergic reactions, not IgE-mediated allergic response. The reaction was stopped by cooling in an ice bath for 10 min. The 24-well plate was centrifuged at 200 g for 5 min at 4 °C, and then 50 μL aliquots of the supernatant were transferred to 96-well plates. To determine the total β-hexosaminidase content, the cells were lysed with 0.1% Triton X-100 in Siraganian buffer (119 mM NaCl, 5mM KCl, 5.6mM Glucose, 1mM $CaCl_2$, 0.1% BSA, 0.4mM $MgCl_2$, 25 mM PIPES, pH. 7.2). The β-hexosaminidase released into the supernatants and in the cell lysates was mixed with an equal volume (50 μL) of substrate solution (p-nitrophenyl-N-acetyl-β-D-glycosamide in 0.1 M citric acid/sodium citrate buffer, pH 4.5) for 90 min at 37 °C. The reaction was stopped by the addition of stop buffer (0.1 M sodium carbonate/sodium bicarbonate, pH 10.5). The percentage of β-hexosaminidase release was assessed by measuring the absorption of the samples at 405 nm with a microplate spectrophotometer, and was calculated as follows: absorbance of culture supernatant at 405 nm × 100/absorbance of total lysate supernatant at 405 nm. C48/80 (30 μg/mL) was used as a positive control.

2.4. Calcium influx assay

One day before calcium influx assay, cell was detached using trypsin from the culture flask, and seeded 96-well black well plates with clear bottom (8,000 ~ 10,000 cells per well), cultured in medium supplemented with DEME supplemented with FBS and puromicin. On the day of calcium influx assay, cell was washed using DPBS and incubated with antagonists for 10 min, and followed by incubation with calcium binding dye under the manufacturer's protocol. (Fluo-4, Thermofisher, USA) The agonist (retinol) was added to the cells and observed under fluorescent microscopy in real-time. The video data was collected, and the fluorescence of 50~100 cells was analyzed by analytical software.

2.5. Self-evaluation index

In the previous numerous studies, irritated skin conditions has been graded according to the criteria proposed by Frosh and Kligman [5] and CTFA guidelines which classifies the irritation (specifically erythema) in to 5 grades: 0 = no reaction, 1 = slight erythema, spotty or diffuse, 2 = moderate uniform erythema, 3 = intense erythema with edema, 4 = intense erythema with edema and vesicles. However, retinoid-induced irritation triggers extremely wide range of irritation-types and severity depending on race, ethnicity, sex, and even each individual, which traditional irritation-measuring guideline does not effectively qualify or quantify these irritations. By that reason, we re-design the self-evaluation guideline for skin irritation as shown in table S1. This self-evaluation was performed in the small pilot study and 2nd large-scale clinical evaluation.

Table S1. Self-evaluation index for retinol-induced irritation. To investigate the diverse type of retinol-induced irritation, the new scoring index was developed.

	**0**	**1**<br>**MILD**	**2**<br>**MODERATE**	**3**<br>**INTENSE**
Desquamation	-	Slightly exfoliated, while other people cannot recognize	Apparently exfoliation observed. Easily exfoliated by external friction or rubbing	Curled corneous tissue observed. Spontaneous exfoliation
Pruritus	-	Feeling slight pruritus, but cannot perceive without concentration	Feeling intermittently moderate pruritus	Intense pruritus. Unconsciously scratch an itchy spot
Burning	-	Very slight burning sensation, but cannot perceive without concentration	Burning sensation as touching warm water	Burning sensation such like a first –degree burn or touching hot material
Dryness	-	Very slight dryness, but cannot perceive without concentration	During facial expression, feeling kinds of "tightness" on the applied area	Intense dryness, such as no use of moisturizer after washing the face.
Stinging *	-	Slightly recognize stinging, but cannot perceive without concentration. Not significant difference when toughing the ROI.	More sensitively recognize the irritations than usual. (Threshold of irritation ↓). When touching the ROI, feels the "stinging irritations", or "chafing-like" irritation.	Recognize the stinging-like irritations under relaxation. When any cosmetics are applied on the ROI, or clothes and fingers are touched on the ROI, recognizes the intense irritations.

* including pricking, sore, and any non-specific irritation.
ROI: region of interest.

Table S2. List of candidate genes used in the genetic analysis and known functions related to skin sensitivity.

Gene	Function (related to skin sensitivity)	Reference
CD86	Treating toxic chemical compounds upregulated CD86 expression.	[6,7]
EGFR	EGFR inhibitor induced cutaneous toxicity.	[6,8]
CD36	Patch testing with SLS increased expression of CD36 in the stratum granulosum. CD36 is a mediator of inflammation in allergic contact dermatitis and irritant contact dermatitis.	[6,9,10]
CD44	CD44 is highly expressed in irritant and allergic contact dermatitis and is required for epidermal permeability barrier homeostasis and keratinocyte differentiation. Topical retinyl retinoate increased CD44 expression.	[6,11–13]
MMP10	Matrix metalloproteinase (MMP2, MMP3, MMP8, MMP10, and MMP13) were up-regulated by UV exposure. MMP10 transcripts were significantly elevated in the rIL-31-treated skin as compared to saline treatment.	[6,14,15]
MMP13	MMP13 transcript abundance was lower in allergic contact dermatitis and in irritant contact dermatitis MMP13 is regarded as a general inflammatory marker	[6,16,17]
IL18	IL18 seems to be implicated in allergic response. Cytokine-producing genes including IL18 have been postulated to influence immunologic functions that are important in the development of atopy. IL18 may play a more central role in the cutaneous immune response. IL18 is constitutively expressed by human keratinocytes. IL18 was used as sensitization marker in a previous study.	[6,18–20]
IL4R	Dermatitis is often associated with a markedly increased concentration of serum IgE and IL18. IL4R is upregulated in allergy disease patients.	[6,21,22]
BCL2	In RA-treated melanocytes and keratinocyte-melanocyte cocultures, S100B expression levels increased more than twofold, and S100B knock-down significantly increased activation of caspase-3, but decreased expression of Bcl-2. Dysregulation of T-cell apoptosis, as a consequence of bcl2 overexpression, was also postulated to contribute to chronicity of inflammation in various cutaneous diseases BCL2 is overexpressed in the lymphocytes of psoriatic skin.	[6,23–25]
COL6A2	Three alpha chains of type VI collagen found in connective tissue Regulates dermal matrix assembly, fibroblast motility, tissue remodeling and wound healing Defect results in keloid formation and abnormal skin phenotypes	[6,26–29]
RARB	Retinoic acid receptor located in the nucleus that act as a transcription factor	[30,31]
RARG	Retinoic acid receptor located in the nucleus that act as a transcription factor Retinoic acid receptor gamma mediates topical retinoid irritation in animal models	[30–32]
RXRB	Retinoic acid receptor located in the nucleus that act as a transcription factor	[30,31]
CCL2	CCL2 expression is increased upon retinol treatment	[33]

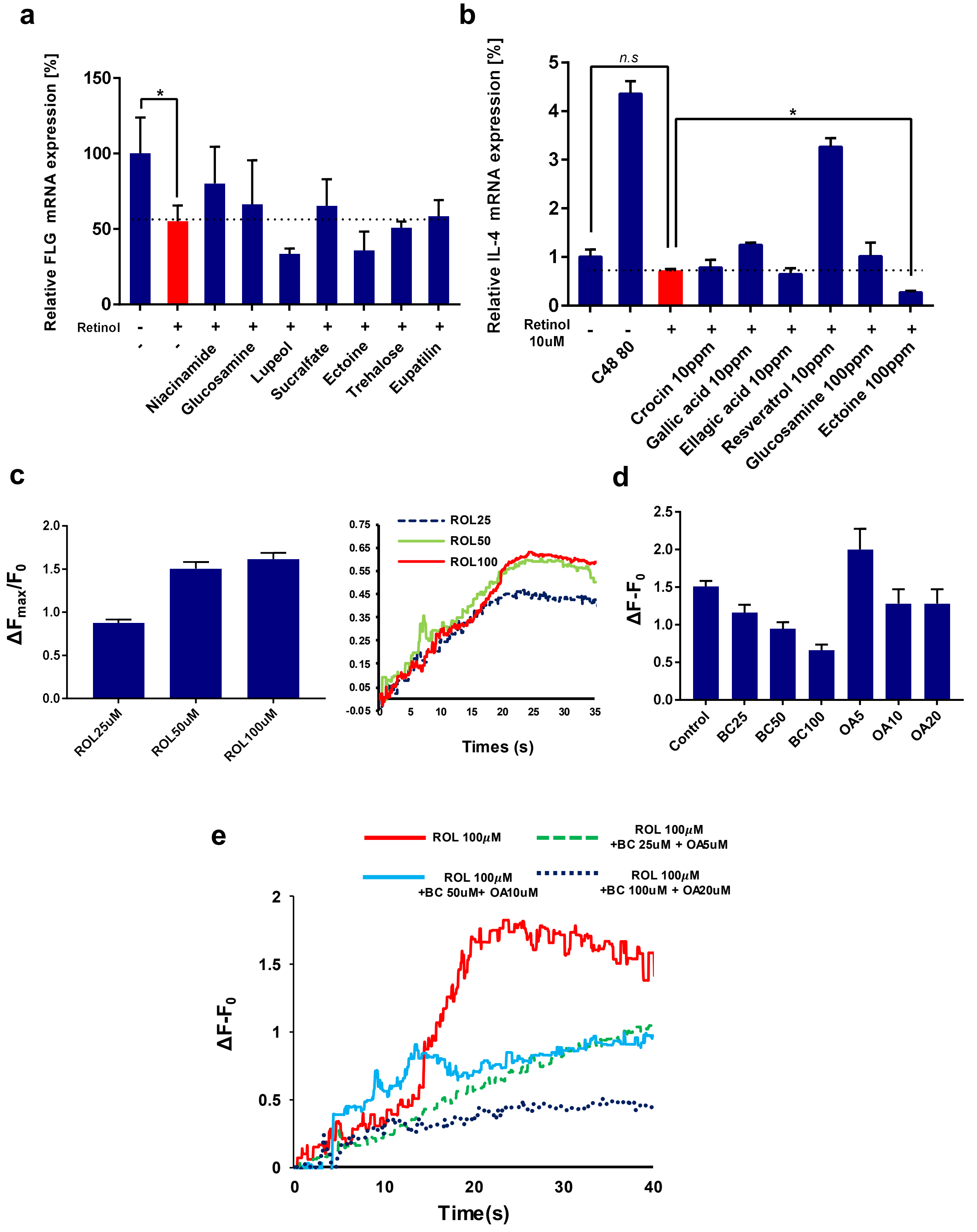

Figure S1. *In-vitro* experiment to screen anti-irritants which could modulate irritation-associated molecular pathogenesis. a) Investigation for skin barrier disruption-associated molecular pathogenesis. Relative mRNA expression of FLG. Keratinocyte HaCat was experimented. Retinol 4ppm was treated. b) Investigation for inflammation (mast cell driven)-associated molecular pathogenesis. relative mRNA expression of IL-4 when RBL-2H3 was treated with 10μM retinol and various candidates. c) Neurogenic inflammation mediated with TRPV1 induced by retinol and d, e) antagonistic effect by 4-t-butylcyclohexanol(BC) and omega-9(OA). * $p < 0.05$; n.s, not significant.

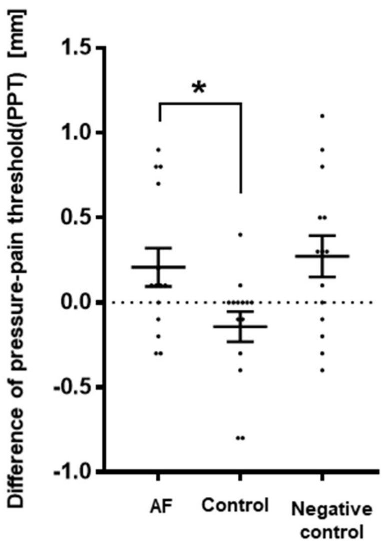

Figure S2. Pressure pain threshold (PPT) measured algometer whose probe has the diameter of 1mm. Control refers to retinol-treated area without AF. Negative control refers to non-treated area. * $p < 0.05$.

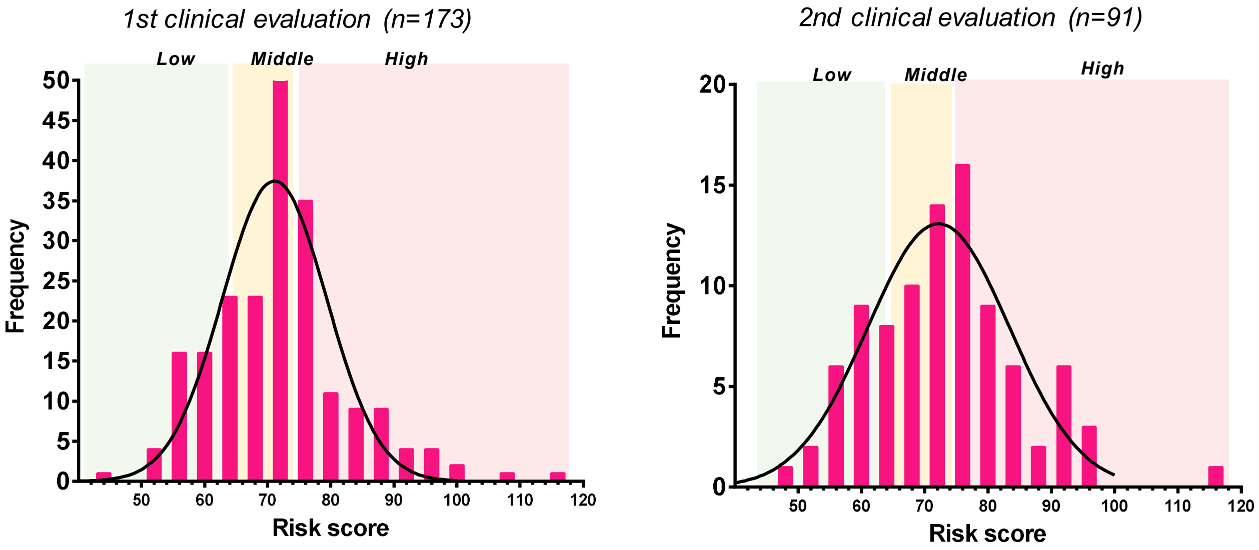

Figure S3. Histogram for risk score on test individuals. 1st clinical evaluation with the aim to investigate to disclose the genetic marker which is regarded to retinol-induced irritation. The 173 people were tested and analyzed. Retinol cream without AF were given. (Left panel) 2nd clinical evaluation with the aim to validate the prediction model for retinol-induced irritation. Retinol cream with AF were given. The test subjects were divided into three groups with following criteria; Low (≤ 65), Middle (65~75), High (75<) (Right panel).

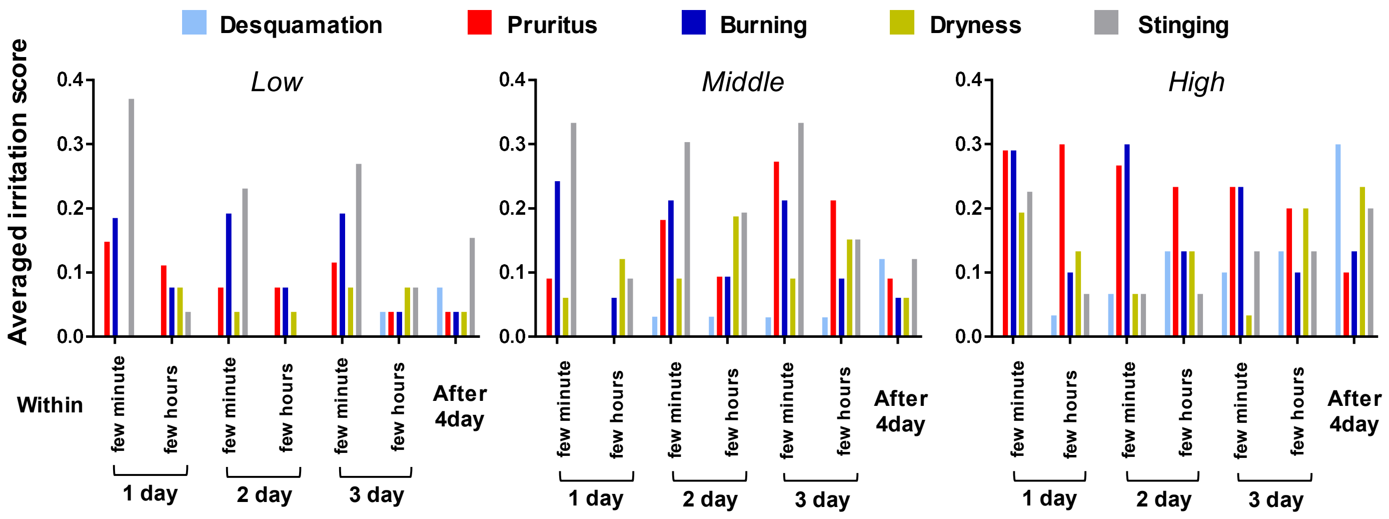

Figure S4. Patterns of irritation in each risk score group. Each type of irritation was averaged.

References

1. Misery, L.; Myon, E.; Martin, N.; Consoli, S.; Boussetta, S.; Nocera, T.; Taieb, C. Sensitive skin: Psychological effects and seasonal changes. *J. Eur. Acad. Dermatol. Venereol.* **2007**, *21*, 620–628.
2. Kim, Y.R.; Cheon, H.I.; Misery, L.; Taieb, C.; Lee, Y.W. Sensitive skin in Korean population: An epidemiological approach. *Ski. Res. Technol.* **2017**, *24*, 229–234.
3. Farage, M.A.; Miller, K.W.; Wippel, A.M.; Berardesca, E.; Misery, L.; Maibach, H. Sensitive skin in the United States: Survey of regional differences. *Family Med. Medical Sci. Res.* **2013**, *2*, https://doi.org/10.4172/2327-4972.100011.
4. Zheng, Y.; Che, D.; Peng, B.; Hao, Y.; Zhang, X.; He, L.; Geng, S. All-trans-retinoic acid activated mast cells via Mas-related G-protein-coupled receptor-X2 in retinoid dermatitis. *Contact Dermat.* **2019**, *81*, 184–193.
5. Frosch, P.J.; Kligman, A.M. The soap chamber test: A new method for assessing the irritancy of soaps. *J. Am. Acad. Dermatol.* **1979**, *1*, 35–41.
6. Cottrez, F.; Boitel, E.; Auriault, C.; Aeby, P.; Groux, H. Genes specifically modulated in sensitized skins allow the detection of sensitizers in a reconstructed human skin model. Development of the SENS-IS assay. *Toxicol. Vitr.* **2015**, *29*, 787–802.
7. Hulette, B.C.; Ryan, C.A.; Gildea, L.A.; Gerberick, G.F. Relationship of CD86 surface marker expression and cytotoxicity on dendritic cells exposed to chemical allergen. *Toxicol. Appl. Pharmacol.* **2005**, *209*, 159–166.
8. Beech, J.; Germetaki, T.; Judge, M.; Paton, N.; Collins, J.; Garbutt, A.; Braun, M.; Fenwick, J.; Saunders, M.P. Management and grading of EGFR inhibitor-induced cutaneous toxicity. *Futur. Oncol.* **2018**, *14*, 2531–2541.
9. Berardesca, E.; Distante, F. The modulation of skin irritation. *Contact Dermat.* **1994**, *31*, 281–287.
10. Willis, C.M.; Stephens, C.J.; Wilkinson, J.D. Selective Expression of Immune-Associated Surface Antigens by Keratinocytes in Irritant Contact Dermatitis. *J. Investig. Dermatol.* **1991**, *96*, 505–511.
11. LugoviĆ-MihiĆ, L.; Novak-Bilić, G.; Vučić, M.; Japundžić, I.; Bukvić, I. CD44 expression in human skin: High expression in irritant and allergic contact dermatitis and moderate expression in psoriasis lesions in comparison with healthy controls. *Contact Dermat.* **2020**, *82*, 297–306.
12. Man, M.; Elias, P.M.; Man, W.; Wu, Y.; Bourguignon, L.Y.W.; Feingold, K.R.; Man, M.-Q. The role of CD44 in cutaneous inflammation. *Exp. Dermatol.* **2009**, *18*, 962–968.
13. Kim, J.E.; Kim, B.; Kim, H.; Kim, H.; Lee, J.D.; Kim, H.J.; Choi, K.Y.; Lee, S.H. Retinyl retinoate induces hyaluronan production and less irritation than other retinoids. *J. Dermatol.* **2010**, *37*, 448–454.
14. Singh, B.; Jegga, A.G.; Shanmukhappa, K.S.; Edukulla, R.; Khurana, G.H.; Medvedovic, M.; Dillon, S.R.; Madala, S.K. IL-31-driven skin remodeling involves epidermal cell proliferation and thickening that lead to impaired skin-barrier function. *PLoS ONE* **2016**, *11*, e0161877, https://doi.org/10.1371/journal.pone.0161877.
15. Cho, B.A.; Yoo, S.-K.; Seo, J.-S. Signatures of photo-aging and intrinsic aging in skin were revealed by transcriptome network analysis. *Aging* **2018**, *10*, 1609–1626.
16. Tam, I.; Hill, K.R.; Park, J.M.; Yu, J. Skin tape stripping identifies gene transcript signature associated with allergic contact dermatitis. *Contact Dermat.* **2020**, *84*, 308–316.
17. Liu, B.; Tai, Y.; Liu, B.; Caceres, A.I.; Yin, C.; Jordt, S.-E. Transcriptome profiling reveals Th2 bias and identifies endogenous itch mediators in poison ivy contact dermatitis.. *JCI Insight* **2019**, *5*, https://doi.org/10.1172/jci.insight.124497.
18. Caballero, M.L.; Quirce, S. Identification and practical management of latex allergy in occupational settings. *Expert Rev. Clin. Immunol.* **2015**, *11*, 977–992.

19. Guyard-Nicodème, M.; Gerault, E.; Platteel, M.; Peschard, O.; Veron, W.; Mondon, P.; Pascal, S.; Feuilloley, M.G.J. Development of a multiparametric in vitro model of skin sensitization. *J. Appl. Toxicol.* **2015**, *35*, 48–58.
20. Sundberg, J.P.; Pratt, C.H.; Goodwin, L.P.; Silva, K.A.; Kennedy, V.E.; Potter, C.S.; Dunham, A.; Sundberg, B.A.; HogenEsch, H. Keratinocyte-specific deletion of SHARPIN induces atopic dermatitis-like inflammation in mice. *PLoS ONE* **2020**, *15*, e0235295, https://doi.org/10.1371/journal.pone.0235295.
21. Zhang, H.; Zhang, Q.; Wang, L.; Chen, H.; Li, Y.; Cui, T.; Huang, W.; Zhang, L.; Yan, F.; Wang, L.; et al. Association of IL4R gene polymorphisms with asthma in Chinese populations. *Hum. Mutat.* **2007**, *28*, 1046–1046.
22. Pascual, M.; Roa, S.; Garcia-Sanchez, A.; Sanz, C.; Hernandez-Hernandez, L.; Greally, J.; Lorente, F.; Dávila, I.; Isidoro-García, M. Genome-wide expression profiling of B lymphocytes reveals IL4R increase in allergic asthma. *J. Allergy Clin. Immunol.* **2014**, *134*, 972–975.
23. Cheong, K.A.; Noh, M.; Kim, C.-H.; Lee, A.-Y. S100B as a potential biomarker for the detection of cytotoxicity of melanocytes. *Exp. Dermatol.* **2014**, *23*, 165–171.
24. Yildiz, L.; Bariş, S.; Senturk, N.; Kandemir, B. Overexpression of bcl‐2 in lymphocytes of psoriatic skin. *J. Eur. Acad. Dermatol. Venereol.* **2003**, *17*, 538–540.
25. Orteu, C.H.; Poulter, L.W.; Rustin, M.H.; Sabin, C.; Salmon, M.; Akbar, A.N. The role of apoptosis in the resolution of T cell-mediated cutaneous inflammation.. *J. Immunol.* **1998**, *161*, 1619–1629.
26. Theocharidis, G.; Drymoussi, Z.; Kao, A.P.; Barber, A.; Lee, D.; Braun, K.M.; Connelly, J.T. Type VI Collagen Regulates Dermal Matrix Assembly and Fibroblast Motility. *J. Investig. Dermatol.* **2016**, *136*, 74–83.
27. Naugle, J.E.; Olson, E.R.; Zhang, X.; Mase, S.E.; Pilati, C.F.; Maron, M.B.; Folkesson, H.G.; Horne, W.I.; Doane, K.J.; Meszaros, J.G. Type VI collagen induces cardiac myofibroblast differentiation: Implications for postiAFction remodeling. *Am. J. Physiol. Heart Circ. Physiol.* **2006**, *290*, H323–H330.
28. Oono, T.; Specks, U.; Eckes, B.; Majewski, S.; Hunzelmann, N.; Timpl, R.; Krieg, T. Expression of Type VI Collagen mRNA During Wound Healing. *J. Investig. Dermatol.* **1993**, *100*, 329–334.
29. Peltonen, J.; Hsiao, L.L.; Jaakkola, S.; Sollberg, S.; Aumailley, M.; Timpl, R.; Chu, M.-L.; Uitto, J. Activation of Collagen Gene Expression in Keloids: Co-Localization of Type I and VI Collagen and Transforming Growth Factor-β1 mRNA. *J. Investig. Dermatol.* **1991**, *97*, 240–248.
30. Dattola, A.; Silvestri, M.; Bennardo, L.; Passante, M.; Scali, E.; Patruno, C.; Nisticò, S.P. Role of Vitamins in Skin Health: A Systematic Review. *Curr. Nutr. Rep.* **2020**, *9*, 226–235.
31. Mukherjee, S.; Date, A.; Patravale, V.; Korting, H.C.; Roeder, A.; Weindl, G. Retinoids in the treatment of skin aging: An overview of clinical efficacy and safety. *Clin. Interv. Aging* **2006**, *1*, 327–348.
32. Chen, S.; Ostrowski, J.; Whiting, G.; Roalsvig, T.; Hammer, L.; Currier, S.J.; Honeyman, J.; Kwasniewski, B.; Yu, K.-L.; Sterzycki, R.; et al. Retinoic Acid Receptor Gamma Mediates Topical Retinoid Efficacy and Irritation in Animal Models. *J. Investig. Dermatol.* **1995**, *104*, 779–783.
33. Kim, B.-H.; Lee, Y.-S.; Kang, K.-S. The mechanism of retinol-induced irritation and its application to anti-irritant development. *Toxicology letters* **2003**, *146*, 65–73.
